# Interpopulation variability and adaptive potential for reduced glyphosate sensitivity in *Alopecurus myosuroides*

**DOI:** 10.1111/wre.12264

**Published:** 2017-08-10

**Authors:** L R Davies, P Neve

**Affiliations:** ^1^ School of Life Sciences University of Warwick Wellesbourne UK; ^2^ Agroecology Department Rothamsted Research Harpenden Hertfordshire UK; ^3^ INRA France

**Keywords:** black‐grass, herbicide resistance, recurrent selection, resistance risk, proactive management

## Abstract

Glyphosate use in the United Kingdom has more than doubled in the last 20 years. Much of this increase is driven by efforts to control herbicide resistant weeds, particularly *Alopecurus myosuroides*, prior to crop drilling. There is precedent for evolution of glyphosate resistance in similar situations, raising concerns over the sustainability of glyphosate use in the UK. We used dose–response experiments to examine variation in glyphosate sensitivity amongst 40 field‐collected *A. myosuroides* populations. No populations were resistant to glyphosate, but ED_90_ values ranged between 354 and 610 g a.i. ha^−1^. Five populations had ED_90_ values significantly higher than the unexposed control population collected from a site at Rothamsted Research with no previous glyphosate exposure. Recurrent selection experiments were performed to determine whether variation in glyphosate sensitivity had a heritable basis. Following two rounds of selection, five of six field populations evolved significantly reduced sensitivity to glyphosate, with R/S ratios, based on estimated ED_50_ values, ranging from 1.2 to 1.5. These results confirm that there is a heritable basis to variation in glyphosate sensitivity. The response to selection was modest. Evolved populations were not highly resistant to glyphosate, although some twice‐selected individuals survived recommended field rates. These results do not represent definitive proof of the potential of *A. myosuroides* to evolve glyphosate resistance, although they do indicate caution is needed when considering the sustainability of increased glyphosate use to control this herbicide resistance‐prone species.

## Introduction

Glyphosate is the world's most important herbicide, demonstrating high levels of efficacy against a broad spectrum of plants, efficient uptake and translocation and a relatively benign toxicological and environmental profile (Duke & Powles, 2008). The first cases of evolved resistance to glyphosate were reported in Australian populations of *Lolium rigidum* Gaudin (rigid ryegrass) in the late 1990s, over 20 years after its commercial introduction (Powles *et al*., 1998; Pratley *et al*., 1999). Since these reports, there has been a steady and sustained increase in the number of weed species evolving resistance to glyphosate. To 2016, glyphosate resistance has been reported in 34 species across six continents (Heap, 2016), although no glyphosate resistance is currently reported in the United Kingdom.

Early cases of glyphosate resistance were associated with its use for season‐long weed control in orchards (Powles *et al*., 1998), or in arable systems where glyphosate was used for broad‐spectrum weed control prior to crop sowing (Pratley *et al*., 1999). The first instance of evolved glyphosate resistance associated with the cultivation of genetically modified glyphosate resistant crops was reported in the USA in 2001 (Van Gessel, 2001). The extensive adoption and over‐reliance on this technology has resulted in evolution of glyphosate resistance in at least a further ten species in the USA, and an increasing number of species in South America (Heap, 2016). In Europe, evolution of glyphosate resistance is currently restricted to six species from two genera, *Lolium* sp. and *Conyza* sp., and has been predominantly associated with glyphosate use for vegetation control in orchard and vine crops. However, the first case of evolution of glyphosate resistance associated with glyphosate use in cereal crops has been reported in Italy (Collavo & Sattin, 2014), where glyphosate was used repeatedly and at low doses (360 g a.i. ha^−1^).

Glyphosate use in UK cereal crops has significantly increased over the period that resistance to glyphosate has evolved in global cropping systems. In the mid‐ to late 1990s, annual use in cereal crops in the UK was below 350 000 kg (applied to 400 000 ha). In 2014, 970 000 kg of glyphosate was applied to almost 1.3 million ha of cereal crops (Garthwaite *et al*., 2015). Increased glyphosate use has resulted, at least in part, from escalating resistance to post‐emergence herbicides in *Alopecurus myosuroides* Huds. (black‐grass) and *Lolium multiflorum* Lam. (Italian ryegrass) (Hull *et al*., 2014). In response, UK farmers have adopted delayed crop sowing and stale seedbeds to encourage early weed germination and emergence, with early emerging weeds being controlled with glyphosate before crop sowing. Modelling studies have identified increased glyphosate use on stale seedbeds, often in systems with reduced or zero tillage, as a major driver for evolution of glyphosate resistance in Australian populations of *L. rigidum* (Neve *et al*., 2003).

A number of glyphosate resistance mechanisms have been identified, including single nucleotide substitutions within the target 5‐enolpyruvylshikimate‐3‐phosphate synthase (EPSPS) gene (target site mutations), vacuolar sequestration and reduced translocation, and EPSPS gene amplification (Sammons & Gaines, 2014). Target site mutations are inherited as single gene traits. The underlying genetics of sequestration‐based mechanisms have not been fully resolved, but are likely to be inherited as quantitative traits. Gene amplification mechanisms also display quantitative patterns of inheritance (Chandi *et al*., 2012; Mohseni‐Moghadam *et al*., 2013), with evidence of a strong maternal component to inheritance in some glyphosate resistant populations (Ribeiro *et al*., 2014).

Most studies to confirm evolution of herbicide resistance, or to monitor the distribution and spread of resistance, adopt a reactive approach. Resistance is suspected following a reduction in field performance; seeds are collected and resistance is confirmed through bioassay (see Burgos *et al*., 2013). Subsequent studies aim to determine the mechanisms, inheritance and fitness correlates of resistance traits. The rationale for these approaches rests on the (sometimes justified) assumption that resistance traits are initially very rare in weed populations, and can only be detected once the frequency of resistance phenotypes increases to levels where resistance becomes evident through reduced efficacy at field doses (see Soteres & Peterson, 2015). This view of the epidemiology and evolutionary dynamics of herbicide resistance neglects the possibility that trait values for quantitatively inherited traits may gradually increase under selection, a phenomenon referred to as ‘creeping resistance’ by Gressel (2002). This phenomenon presents testable hypotheses that may enable a more proactive approach to assessing risks associated with emerging herbicide resistance threats.

Given (i) widespread resistance to multiple herbicide modes of action in UK populations of *A. myosuroides*, (ii) the propensity of this outcrossing species to evolve resistance to herbicides, (iii) increased use of glyphosate in the UK and (iv) a global precedent for the evolution of glyphosate resistance, there is a pressing need for proactive studies to explore the risks of glyphosate resistance in this species. Here, we report dose–response and experimental evolutionary approaches that test two hypotheses, relating to the evolution of glyphosate resistance in *A. myosuroides*: (i) there are significant differences in sensitivity to glyphosate amongst *A. myosuroides* populations on arable farms in the UK and (ii) recurrent selection with glyphosate will result in further reductions in glyphosate sensitivity. Together, testing these hypotheses enables us to begin to evaluate the potential for evolution of glyphosate resistance in *A. myosuroides* and to establish a baseline against which future changes in sensitivity and the possible evolution of field resistance can be assessed.

## Materials and methods

### Dose–response bioassays to establish glyphosate sensitivity

#### Plant material

**Figure 1:**
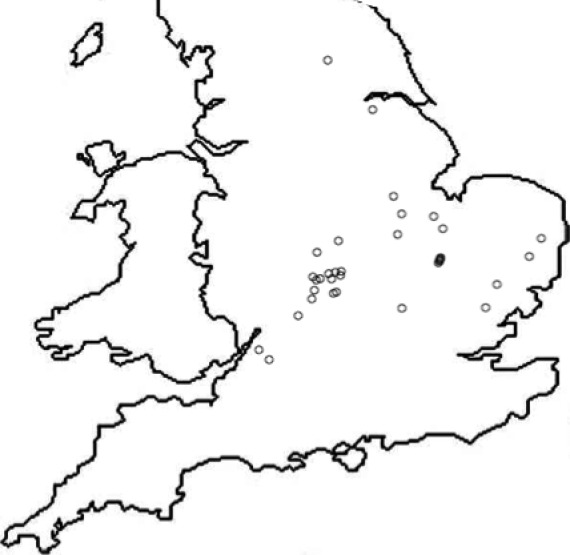
Collection sites of 40 UK *Alopecurus myosuroides* populations collected in July 2012 and tested for glyphosate sensitivity using dose–response analysis.

In July 2012, seed samples were collected from 32 *A. myosuroides* populations from a series of arable farms throughout the main range of the species in England (Fig. 1). A population was defined as all *A. myosuroides* plants growing in a single field and seeds were collected from one field per farm. Collection sites were chosen based on the presence of *A. myosuroides*, and not on prior knowledge of glyphosate use history within those fields. A further seven populations were provided directly by ADAS from their collections. Populations are named (e.g. AHE112) accordingly using a standard nomenclature with A representing *A. myosuroides*, the following two letters identifying the English county in which the population was collected (i.e. HE = Hertfordshire), and 112 representing population number 1 from Hertfordshire, collected in 2012. One population was collected from a plot at the Broadbalk experiment at Rothamsted Research that had no previous exposure to herbicides (AHE112) (Moss *et al*., 2004); this population was used as a reference sensitive population in analyses. The AES112 population (Peldon) has been studied extensively and exhibits broad‐spectrum, non‐target site‐based resistance to herbicides (Hall *et al*., 1997; Cummins *et al*., 2013). A total of 40 populations were included in initial dose–response experiments.

At the 32 sampling sites, seeds were taken from between 130 and 531 seed heads (mean: 375) per population, with mature seed harvested by rubbing seed heads into a paper bag. To ensure a representative population sample, seeds were collected by traversing each field along a W‐shaped transect, collecting seeds from mature plants at 0.5 m intervals in heavily infested fields or from every plant on the transect in less heavily infested fields. After collection, seeds were dried and stored in paper bags at 15% RH, 15*°*C. After drying, seed lots were threshed and cleaned to remove unfilled seeds and debris.

#### Dose–response protocol

Following drying, threshing and cleaning, the 40 seed populations were placed in an incubator at 30*°*C for 6 weeks to break seed dormancy. At commencement of dose–response experiments in October 2012, seeds were sown in a regular pattern, 5 mm below the soil surface, with two seeds sown at each of eight positions in 90 × 90 × 90 mm square plastic pots. Pots contained a 2:1:1 mixture of topsoil (English loam blended with organic matter and nutrients, pH: 6.5–7.5), Levington's M2 compost (pH: 5.5–6, N: 200, P: 150, K: 200 mg L^−1^) and silver sand (lime‐free washed silica sand). Following seed sowing, pots were thoroughly watered and were placed in a glasshouse compartment with a 17 h day length and supplementary lighting. Temperature was set to 20*°*C with venting at 22*°*C during daylight hours, and 12*°*C with venting at 15*°*C during darkness. Pots were watered from above as required throughout the experiment.

The dose–response design consisted of eight glyphosate doses (0, 81, 162, 270, 405, 540, 810 and 1080 g a.i. ha^−1^), 540 g a.i. ha^−1^ being the recommended dose for *A. myosuroides* control in the UK. There were five replicate pots per population by dose combination. Pots were arranged as subplots (trays) by dose for ease of herbicide application. There were two trays per dose by block combination with the 40 *A. myosuroides* populations randomly allocated to positions within the two trays. There were 16 trays per block (eight doses with two trays per dose) and pairs of trays were completely randomised within blocks within the glasshouse.

Seeds were sown by block over consecutive days. Eighteen days after sowing, the number of seedlings per pot was thinned to eight (one per sowing location in each pot), ensuring that remaining plants were a similar size (2–4 leaves) at the time of herbicide application. Glyphosate [Roundup ProBiactive, 360 g L^−1^ glyphosate present as the isopropylamine salt at 480 g L^−1^ (41.1% w/w) (Monsanto)] was applied with an experimental track sprayer (generation III research sprayer, DeVries) using a flat fan nozzle (FE80/0.8/3) calibrated to deliver 200 L ha^−1^ at a pressure of 300 KPa. Glyphosate was applied by block 24–28 days after sowing (blocks 1 and 3–28 days, blocks 2 and 4–27 days, block 5–24 days after sowing). Following herbicide application, plants were returned to the glasshouse. Plant survival was assessed 28 days after herbicide application.

### Glyphosate low‐dose selection experiments

#### Plant material

In order to determine the potential to select for reduced glyphosate sensitivity under recurrent glasshouse selection, six populations were chosen for further glyphosate selection experiments. Based on per cent survival at 270 g ha^−1^ (half recommended field rate), four populations [ACA212 (71%), ACA412 (68%), AES112 (67%) and AES212 (71%)] were chosen to represent reduced levels of glyphosate sensitivity from amongst the original collection, one population [AWA712 (57%)] exhibited intermediate sensitivity, and one population [ASF112 (51%)] was sensitive.

#### Selection protocol generation 1

In December 2012, seed populations were placed in an incubator at 30*°*C for 6 weeks to break seed dormancy. After dormancy breaking, two seeds were sown into each of 357 wells in plant propagation trays (http://containerwise.co.uk/propagation-trays-600-400.html). The trays were filled with a 2:1:1 mix of topsoil, compost and sand as previously described. A total of 12 trays were sown, two treatments for each of the six populations, one glyphosate‐selected tray and one control tray that was not exposed to glyphosate selection. Trays were arranged in a completely random design in a glasshouse compartment and were watered as required over the course of the experiment. Twenty‐five days after sowing, the number of seedlings per tray was thinned to 150, ensuring that all remaining plants were a similar size (2–4 leaves) at the time of herbicide application. Four days after thinning, glyphosate was applied to treated trays at a rate of 405 g a.i. ha^−1^ using a Berthoud knapsack sprayer with a flat fan, even spray nozzle (FE80/0.8/3) calibrated to deliver 200 L ha^−1^ at a pressure of 300 KPa. Based on previous dose–response experiments, this glyphosate selection dose was chosen to provide *c*. 60–80% mortality of treated plants. No glyphosate was applied to control trays and all trays were returned to the glasshouse after treatment.

Mortality of treated plants was assessed 21 days after spraying and was lower than anticipated based on previous dose–response results (30–40%). For this reason, surviving plants were cut 10 mm above soil height and were allowed to regrow for a period of 10 days prior to reapplication of glyphosate at 405 g a.i. ha^−1^ as described above. Mortality was reassessed 21 days after the second glyphosate treatment and survivors were repotted into 15 cm pots containing potting mixture, 5–7 plants per pot. Thirty‐two survivors were repotted for ACA212, 49 for ACA412, 69 for AES112, 52 for AES212, 35 for ASF112 and 56 for AWA712. An identical number of plants from untreated control lines were randomly selected and repotted for each seed population. Pots for each glyphosate‐treated and untreated line were moved to polythene tunnels and placed in pollen cages with one population per cage. Isolation of plants in pollen‐proof enclosures enabled bulk crossing (panmixis) between plants within a selection line and prevented cross‐pollination between selection lines. Plants were grown to maturity in ambient conditions, producing a single seed population for each selection line. Seeds were collected as they matured and dried and stored in paper bags at 15% RH, 15*°*C.

#### Selection protocol generation 2

In February 2014, seed dormancy of selection lines was broken. Seed populations were sown in propagation trays, maintained in the glasshouse and thinned to 150 evenly sized individuals as described above. Glyphosate was applied at 360 g a.i. ha^−1^ using a track sprayer calibrated to deliver 200 L ha^−1^ at 300 KPa. Twenty‐three days after treatment, plants were assessed for mortality. Survivors were repotted into 15‐cm pots containing standard potting mixture, 5–7 plants per pot, and moved to pollen cages to mature and produce seed. For ACA212, 31 survivors were repotted, 24 for ACA412, 40 for AES112, 50 for AES212, 45 for ASF112 and 20 for AWA712. Seed was harvested and stored as described above.

### Glyphosate dose–response experiment to assess response to selection

Following completion of two rounds of recurrent glyphosate selection, a glyphosate dose–response experiment was performed to quantify responses to glyphosate selection. Eighteen seed populations were included; three selection lines (control unselected and first‐ and second‐generation glyphosate‐selected) for each of the six *A. myosuroides* populations. Seed dormancy was broken prior to establishment of experiments. In November 2014, 100 seeds per selection line were sown into 90 mm Petri dishes containing three 85 mm filter papers and 5 mL of deionised water. Petri dishes were sealed with parafilm and incubated for 7 days at an alternating 23*°*C (light)/9*°*C (dark) temperature regime with a 12 h photoperiod to promote seed germination. Eight germinated seedlings were transplanted in a regular pattern into 90 × 90 × 90 mm square plastic pots containing standard potting mixture. Pots were maintained with regular watering in a glasshouse compartment as previously described.

The dose–response design consisted of seven glyphosate doses (0, 81, 162, 270, 405, 540 and 810 g a.i. ha^−1^). There were 18 selected lines in total, three for each population: the control/unselected line (C2), and the lines selected with glyphosate for one (T1) and two (T2) generations. There were four replicate pots selection line by dose combination, resulting in a total of 504 pots. Following seedling transplanting, individual pots were randomly arranged in trays, 18 pots per tray, with one tray representing a glyphosate dose by block (replicate) combination. Pots were arranged in this way (as subplots) for ease of herbicide application. There were seven trays per block (seven doses with one tray per dose), and trays were completely randomised within blocks within the glasshouse.

Ten days after transplanting, seedlings were thinned to provide six evenly sized individuals per pot. Glyphosate was applied 14 days after transplanting with a track sprayer as previously described. Following herbicide application, plants were returned to the glasshouse. Plant assessments were performed 21 days after herbicide application. The number of surviving plants per pot was recorded and above‐ground plant biomass was harvested.

### Data analysis

Results were analysed by nonlinear regression using the DRC package in R (version 2.15.3). Survival data for glyphosate dose–response experiments was modelled by fitting 2‐parameter sigmoidal functions with binomial endpoints. Two‐parameter models provided estimates for ‘*e*’, the inflection point of the model, and ‘*b*’, the slope of the dose–response curve around the inflection point. All data series were fitted to log‐logistic and Weibull functions and lack‐of‐fit tests were performed to determine the most appropriate function for individual data sets. In models assuming a log‐logistic distribution, the *e* parameter is equal to the ED_50_, the dose resulting in a 50% reduction is the response variable. For the asymmetric Weibull distribution, *e* is not equivalent to ED_50_ and this value is calculated. In cases where all models demonstrated some lack‐of‐fit, the best fitting model was selected (Ritz, 2010).

For model simplification and to determine whether there were significant differences in model parameters between populations and selection lines, a series of 2‐parameter models were fitted in DRC with constrained slope (*b*) and effective dose (*e*) parameters. For example, where the slope was constrained, all populations were modelled with the same slope parameter, such that differences between populations were explained solely by variation in the effective dose parameter (and vice versa where the *e* parameter alone was constrained). Constrained models were compared to the full (unconstrained) model using a lack‐of‐fit *F*‐test to determine whether there was a significant difference between constrained and full models. For the initial dose–response experiment, a log‐logistic (Ritz, 2010) 2‐parameter function with a constrained *e* parameter and unconstrained *b* parameter provided the best model fit for survival data, indicating that population ED_50_ values were not significantly different and that differences between populations were the result of significantly different dose–response slopes. For the experiment to explore dose–responses of the glyphosate‐selected lines, data were modelled with a 2‐parameter Weibull‐1 (Ritz, 2010) function with a constrained slope (*b*) and unconstrained *e* parameter, such that population differences were explained by different ED_50_ parameter estimates. Following model selection and simplification, ED_50_ and ED_90_ values for survival data were calculated. The SI function in the drc package was used to determine significant differences between estimated ED values (Ritz *et al*., 2015) to determine whether any populations had significantly reduced sensitivity in comparison with the reference AHE112 population, and to establish whether there had been a significant response to glyphosate selection in the selection experiments.

## Results

### Variation in glyphosate sensitivity amongst 40 UK *A. myosuroides* populations

**Figure 2:**
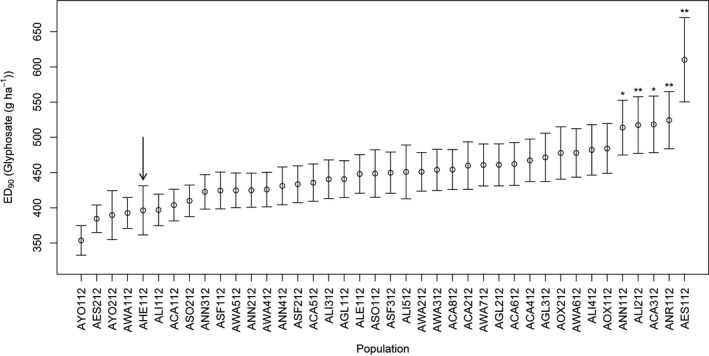
Calculated ED_90_ values from log‐logistic dose–response models for 40 field‐collected *Alopecurus myosuroides* populations from the UK. Error bars are standard errors of ED_90_ parameter estimates. Significant differences between the previously unexposed Rothamsted population (AHE112, shown with an arrow) and farm‐collected populations are indicated (*P <* *0.05, **P <* *0.01).

Dose–response model fitting indicated there was significant variance amongst populations in slope, but not ED_50_. This confirms that there were significant differences between populations in response to glyphosate. Calculated ED_50_ values were constrained to 280 (±2.34) g ha^−1^ and ED_90_ values ranged between 354 and 610 g ha^−1^. Hence, the ratio of ED_90_ values between the least and most sensitive populations was 1.7. Five populations had significantly higher ED_90_ values (significant SI in drc package) than the reference susceptible population, AHE112. These populations were ANR112 (*P *=* *0.006), AES112 (*P *=* *0.008), ALI212 (*P *=* *0.009), ANN112 (*P *=* *0.011) and ACA312 (*P *=* *0.044) (Fig. 2). The SI function in drc does not correct for multiple comparisons and a more conservative test of significantly less sensitive populations would likely exclude ACA312. Thirty‐seven of the 40 populations exhibited >90% control by glyphosate at the recommended field rate of 540 g a.i. ha^−1^. On the basis of these results, we conclude that whilst there is significant variation in sensitivity between populations, no evolved resistance to glyphosate was identified, as surviving plants in the least sensitive populations (AES112, ALI212 and ACA212) were severely impacted by glyphosate treatment. There was no correlation between population size (using number of seed heads collected as a proxy for population size) and the estimated glyphosate ED_90_ value (Pearson's correlation; *P *=* *0.211). When we grouped population by county, there were no significant differences in dose–response curves amongst counties, indicating that geography was not a strong indicator of glyphosate sensitivity (*P *=* *0.87). However, it was notable that one of the most sensitive populations was AHE112, a population sampled from plots at the Broadbalk experiment at Rothamsted Research that had no history of herbicide exposure. Similarly, it is intriguing that AES112, a population in which non‐target site resistance was confirmed in 1982 and that has subsequently been exposed to intense selection with glyphosate, exhibited considerably reduced sensitivity to glyphosate. It was not possible to obtain detailed glyphosate exposure histories for sampled populations, and therefore, we cannot definitively conclude that differences in sensitivity result from contrasting glyphosate use histories. Selection experiments were performed in order to determine whether field‐collected *A. myosuroides* populations would respond to recurrent selection with glyphosate in the glasshouse. These experiments aimed to determine whether variation in glyphosate sensitivity has a heritable basis and may be indicative of future potential for evolution of reduced sensitivity to glyphosate in this species.

### Response to recurrent glyphosate selection

For the first generation of selection, survival at the selecting dose (405 g a.i. ha^−1^) varied between 21% and 46% (ACA212 21%, ACA412 33%, AES112 46%, AES212 35%, ASF112 23% and AWA712 37%). For the second generation of selection, survival varied between 13% and 33% (ACA212 21%, ACA412 16%, AES112 27%, AES212 33%, ASF112 30% and AWA712 13%).

**Figure 3:**
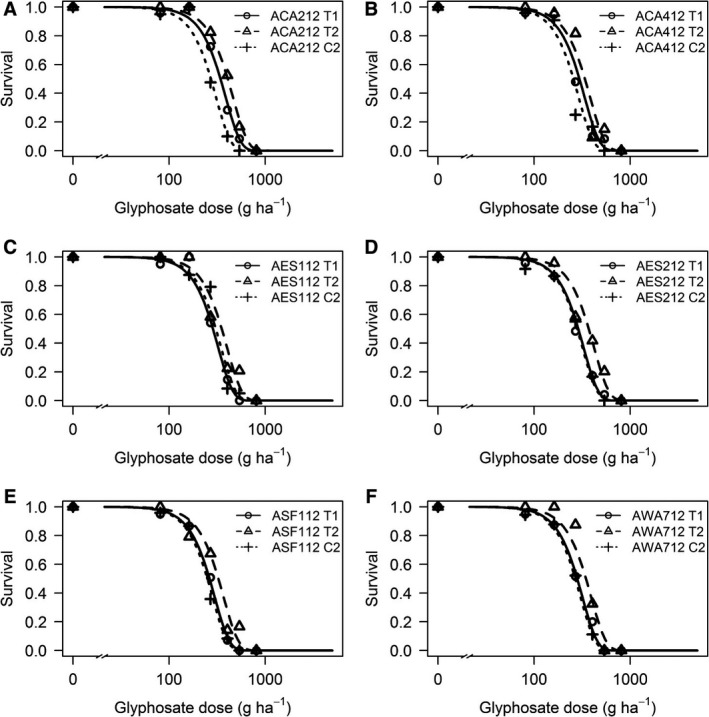
Dose–response curves (Weibull 2‐parameter function) for six *Alopecurus myosuroides* populations subjected to recurrent glyphosate selection (T1, one generation of selection, T2, two generations of selection, C2, control, unselected line). Symbols represent mean observed survival data and lines are fitted regression models. (A) Population ACA212; (B) Population ACA412; (C) Population AES112; (D) Population AES212; (E) Population ASF112; (F) Population AWA712.

**Figure 4:**
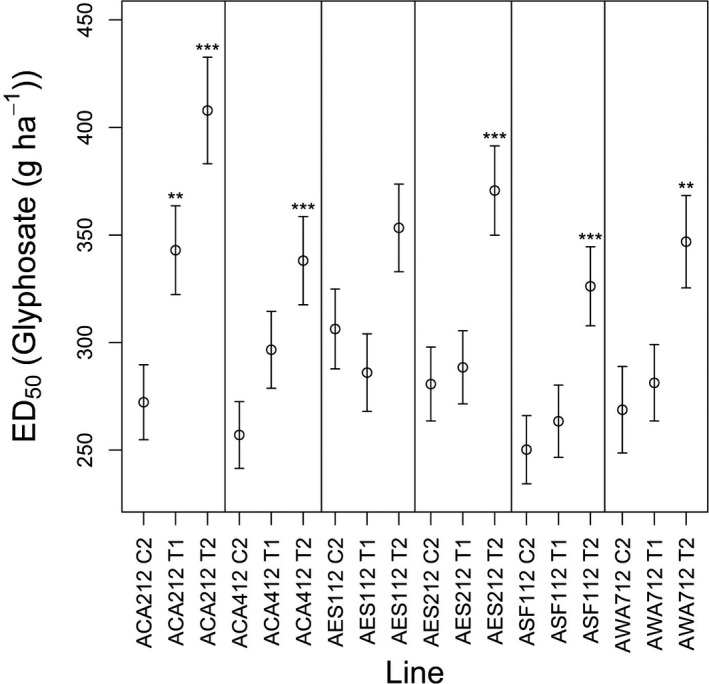
Estimated ED_50_ values for glyphosate‐selected and control *Alopecurus myosuroides* lines. Error bars are standard errors. T‐tests were performed to compare ED_50_ estimates for the control (C2) populations to the once‐selected (T1) and twice‐selected (T2) lines (**P <* *0.01, ***P <* *0.001).

Plant survival data from the dose–response experiment was modelled with a 2‐parameter Weibull‐1 function with a constrained slope (*b*) and unconstrained effective dose (*e*) parameter (Fig. 3). There were significant differences in calculated ED_50_ values between control, unselected lines (C2) and glyphosate‐selected lines (T1 and T2) for five of the six selected populations (Fig. 4). The R/S ratios for T2 and C2 lines ranged between 1.2 and 1.5, indicating that responses to selection were moderate in extent. For five of the six C2 lines, 100% control was achieved at 540 g a.i. ha^−1^ (4% survival was observed for the AES112 C2 line), whilst survival at this dose ranged between 0% and 16% for the twice‐selected (T2) lines (Fig. 3). Whilst these results do not represent evidence for evolved resistance to glyphosate at the population level, they are clearly indicative of a heritable basis for variation in glyphosate sensitivity within *A. myosuroides* populations that can result in plant survival at field doses in glasshouse experiments. The constant slope between the selected lines shows that although selection has decreased glyphosate sensitivity in *A. myosuroides* populations, it has not increased variance for glyphosate response in the selected lines.

## Discussion

Our results identified no glyphosate resistance amongst a random collection of *A. myosuroides* populations from England, although there was significant interpopulation variation in sensitivity. Selection experiments resulted in significant shifts in population‐level response to glyphosate in five of six experimental populations, indicating that variation in glyphosate sensitivity has a heritable basis and highlighting the potential for evolution towards reduced sensitivity under recurrent selection.

A number of previous studies have identified significant variation in glyphosate sensitivity between weed populations that have not evolved resistance. Barroso *et al*. (2010) collected five populations each of *Lolium rigidum* and *Bromus diandrus* Roth (ripgut brome), all with no previous glyphosate exposure, and reported I_50_ values ranging between 17–46 g a.i. ha^−1^ and 85–117 g a.i. ha^−1^ respectively. In Sweden, an order of magnitude difference in GR_50_ values (17–218 g a.i. ha^−1^) was found amongst 69 clones of *Elymus repens* (L) Gould (common couch) collected from across the species range (Espeby *et al*., 2011). In North America, significant variation in mortality of *Oryza sativa* L. (weedy red rice) was reported amongst populations collected in Arkansas (Burgos *et al*., 2011) and GR_50_ values for seven *Ipomoea lacunosa* L. (pitted morning‐glory) populations from the southern United States ranged between 0.65 and 1.23 kg a.i. ha^−1^ (Burke *et al*., 2009). In a study exploring variation in glyphosate sensitivity in 31 populations of *Brassica juncea* (L.) Vassiliĭ Matveievitch Czernajew (Indian mustard) from China, Huangfu *et al*. (2007) reported between 20 and 80% mortality at 337 g a.i. ha^−1^ and 30–90% mortality at 665 g a.i. ha^−1^. Brotherton *et al*. (2007) measured seed production ranging from 6% to 100% of untreated controls amongst 25 accessions of *Arabidopsis thaliana* (L) Heynh.

Although none of these studies were able to establish a clear epidemiological link between glyphosate use history and reductions in glyphosate sensitivity, they nevertheless establish significant variation in glyphosate sensitivity amongst weed species from various parts of the world, similar to that found in *A. myosuroides*. For UK populations of *A. myosuroides*, it is intriguing that one of the most sensitive populations (AHE112) was collected from a site with no previous glyphosate exposure and that one of the most tolerant populations (AES112) was from a site where herbicide resistance was first reported in 1982, and where glyphosate use has been extensive (no long‐term historical records of glyphosate use could be collected, although the land manager recounted that two to three glyphosate applications per growing season had been applied for more than 30 years). The Peldon (AES112) population also has high levels of enhanced metabolism for a number of herbicide modes of action (Hall *et al*., 1997; Cummins *et al*., 2013), and whilst there is currently little evidence to suggest that these mechanisms could confer cross‐resistance to glyphosate, the links between non‐target site resistance and reduced sensitivity to glyphosate are worthy of further investigation. Variation in sensitivity to glyphosate, in response to previous selection, or based on standing pre‐selective genetic variation, may be indicative of the potential for the evolution of reduced sensitivity, and ultimately of population‐level resistance, under recurrent selection.

Experiments based on recurrent selection have previously been used to demonstrate the potential for low herbicide doses to select for resistant phenotypes from standing variation within naïve weed populations (Neve & Powles, 2005; Busi & Powles, 2009; Manalil *et al*., 2011; Busi *et al*., 2012). The same rationale has been used here to explore whether variation in glyphosate sensitivity is heritable, providing the basis for, or being indicative of, the potential for populations to evolve reduced glyphosate sensitivity (‘creeping’ resistance). Intuitively, shifts in sensitivity (RI = 1.2–1.5) are relatively modest, although it is worth stressing that the ED_50_ of selected lines is generally 100 g ha^−1^ glyphosate higher than in unselected lines. These modest, although significant shifts, are the first empirical evidence for a heritable basis for variation in glyphosate sensitivity and they are consistent with field observations that, despite many years of selection, glyphosate resistance has not evolved in *A. myosuroides*. They indicate some genetic potential for evolution of glyphosate resistance in this species, whilst being consistent with the current lack of reports of field‐evolved resistance. Moreover, results were comparable to those of Busi and Powles (2009), who observed a twofold increase in the LD_50_ of a population of *L. rigidum* following three rounds of recurrent glyphosate selection. Field resistance to glyphosate has evolved in *L. rigidum* in Australia (Powles *et al*., 1998) and in Italy (Collavo & Sattin, 2014), often associated with use of low glyphosate doses and so the experimental results of Busi and Powles (2009) correspond with the potential for evolution of glyphosate resistance in the field. The experimental evolution of creeping resistance in multiple *A. myosuroides* populations is not direct evidence of future risks of glyphosate resistance, although results are noteworthy with some individuals capable of surviving current recommended field rates. The current study provides a rare example of proactive studies to explore the potential for evolution of resistance (see Busi *et al*., 2012) and indicates the need to better understand the interactions between frequency of use, dose rate and management on glyphosate resistance risks in *A. myosuroides* in the UK and more widely in Europe.

A number of caveats apply to the current study. Selection experiments, conducted in the glasshouse, maximise the opportunity for less sensitive individuals to cross‐pollinate. In this situation, in an outcrossing species, the minor genes that underpin quantitative differences in glyphosate sensitivity will be recombined efficiently leading to more rapid increases in the resistance phenotype than would occur under field conditions, where surviving reproductive plants will include those that have not been selected with glyphosate, thus diluting selection for resistance. Target site and non‐target site resistance mechanisms have been reported in field‐evolved glyphosate resistant weed populations (Sammons & Gaines, 2014). Target site resistance is often conferred by single nucleotide polymorphisms that result in an amino acid substitution at position 106 of the EPSPS gene. The genetic basis of non‐target site resistance has not been fully resolved, but is likely due to polygenic or quantitative patterns of inheritance. Jander *et al*. (2003) found no target site mutations in the EPSPS gene of 125 000 mutagenised seeds of two *A. thaliana* accessions, suggesting that the frequency of these mutations will be low in weed populations. Our experiments, based on selection in small experimental populations, preclude the possibility of selecting rare target site mutations.

The resistance‐prone grass weed *A. myosuroides* has evolved field resistance to seven herbicide modes of action (Heap, 2016), although no resistance to glyphosate has been reported to date. In closely related species from the genus, *Lolium* sp., the widespread evolution of resistance to post‐emergence herbicides, particularly those with ACCase and ALS modes of actions has increased use of glyphosate, resulting in evolution of glyphosate resistance (Powles *et al*., 1998; Preston *et al*., 2009; Owen & Powles, 2010; Collavo & Sattin, 2014). Escalating levels of resistance to selective modes of action in populations of *A. myosuroides* from England and other countries in northern and western Europe is similarly increasing use of, and reliance on, glyphosate for control of this species, leading to concerns about the potential for evolution of glyphosate resistance in this species. Our results establish potential for *A. myosuroides* to evolve reduced sensitivity to glyphosate under recurrent selection and highlight the importance of ongoing research to better understand the potential for evolution of glyphosate resistance in this species. Ongoing monitoring of changes in glyphosate sensitivity is needed to ensure the continued efficacy of this important herbicide for *A. myosuroides* control and to guard against the threat of evolution of glyphosate resistance.
